# Art Therapy in the Treatment of Traumatic Stress: A Narrative Review

**DOI:** 10.3390/bs16101729

**Published:** 2026-09-23

**Authors:** Jenna Al-Nouri, Holly Feen-Calligan, Lana Ruvolo Grasser

**Affiliations:** 1 Department of Psychiatry and Behavioral Neurosciences, School of Medicine, Wayne State University, Detroit, MI 48201, USA; jalnouri@umich.edu; 2 College of Education, Wayne State University, Detroit, MI 48202, USA; hfeen@wayne.edu; 3 Ben L. Silberstein Institute for Brain Health, Division of Research and Innovation, Wayne State University, Detroit, MI 48201, USA; 4 Department of Psychology, College of Liberal Arts and Sciences, Wayne State University, Detroit, MI 48201, USA

**Keywords:** narrative review, PTSD, mental health, children and adolescents, adults, psychotherapy

## Abstract

Art therapy is a nonverbal form of therapy that can provide individuals with a safe and supportive environment for expression and processing, particularly of trauma-related emotions and memories. For individuals who have experienced trauma, this nonverbal, creative, and tactile modality may serve as a beneficial complement, integrative aspect, or alternative to current gold-standard forms of care, including exposure-based cognitive behavioral therapy, cognitive processing therapy, and pharmacotherapy. In this narrative review, findings across the hierarchy of evidence, from theory and case studies to controlled trials and meta-analyses, in both adults and youth, are summarized to explore the benefits and limitations of art therapy to address trauma and related psychopathology. Findings are contextualized within the Expressive Therapies Continuum (ETC). The majority of available evidence comes from case and cohort studies conducted with small samples of one to 30 individuals per study. Systematic reviews and meta-analyses were largely limited by inconsistencies in providers, protocols, and assessments, as well as a lack of long-term follow-up data to understand the persistent effects of art therapy. While a singular narrative review cannot fully reflect the diversity and richness of art therapy or types of traumatic stress in different societies and situations, this article aims to guide future research, with specific design recommendations, prioritization of randomized-controlled trials, and a call for manualized treatment protocols.

## 1. Introduction

Trauma is pervasive, affecting up to 90% of individuals during the life course. How an individual perceives and processes traumatic events is a critical factor in making meaning out of the traumatic experience and psychophysiological outcomes (Hinchey et al., 2022; Javanbakht et al., 2020; Naff, 2014). Lack of stable, supportive relationships and communities, presence of preexisting or comorbid mental and physical health conditions, and feelings of insecurity, loneliness, or negative self-views can affect the manner in which individuals process traumatic experiences and respond to treatment (Hass-Cohen et al., 2018). Art therapy is a resilience-building strategy that can be implemented in individual or group settings and integrated across ecological systems to aid individuals in reframing their cognitions and emotions surrounding trauma(s) (a shared goal of cognitive behavioral therapies like cognitive processing therapy; Courtois et al., 2017). The Expressive Therapies Continuum (ETC) serves as a framework in which to conceptualize therapeutic experientials leveraged in art therapy on a continuum from kinesthetic-sensory to cognitive symbolic (Kagin & Lusebrink, 1978). As summarized in Grasser and Marusak (2023), the ETC can be understood within the context of the neurobiology underlying emotions and behaviors, ultimately resulting in art therapy driving meaningful changes in psychopathology. For example, kinesthetic-sensory experiential activities engage sensorimotor and limbic brain regions that perceive and encode traumatic events and reminders thereof, while cognitive-symbolic experientials engage prefrontal regions that regulate emotions and behaviors. In addition to examples that are explicitly integrated within the ETC (Hinz, 2019; Lusebrink & Hinz, 2021), readers will be able to situate the individual and group applications described here, especially in studies that do not explicitly identify ETC processes. Readers can begin to think about the intersection of ETC processes with art therapy research related to the treatment of trauma and emotional dysregulation, strengthening both art therapy research and application of the ETC.

Art therapy integrates experientials (i.e., guided therapeutic activities) that probe creativity and adaptability to foster cognitive flexibility (Bronfenbrenner, n.d.; Gilgoff et al., 2020; Minton & Faber, 2016). Art therapy has been used with a range of clinical populations: not only those who have experienced trauma, but also (and not limited to) cancer patients (Geue et al., 2010; Wood et al., 2011), youth and adults with chronic medical conditions (Angheluta & Lee, 2011; Beebe et al., 2010; Councill, 2003; O’Neill & Moss, 2015), persons who are incarcerated (Kõiv & Kaudne, 2015; Koomson et al., 2020; Persons, 2009), the elderly (Gussak & Rosal, 2016), and non-clinical populations who face ongoing daily challenges such as work stress (Huet, 2015). While there have been a number of reviews describing the effectiveness of art therapy (Maujean et al., 2014; Reynolds et al., 2000; Slayton et al., 2010), this review is unique and makes a novel contribution in its focus on the effectiveness of art therapy in trauma-exposed youth and adults (Regev & Cohen-Yatziv, 2018). Previous reviews of art therapy in the trauma treatment space have looked exclusively at adults (Ramirez et al., 2016; Schouten et al., 2015), queried art therapy alongside other methods in broader-scope reviews (Wethington et al., 2008), and focused on cohorts with specific exposure types, like military trauma (Ramirez et al., 2016). The present review is novel in its inclusion of both youth and adults, organization according to the hierarchy of evidence, and integration with the ETC. We highlight the diversity of populations who can be and are treated with art therapy, as well as the supporting evidence for art therapy in reducing trauma-related psychopathology. Two authors independently searched for original research articles on “art therapy” AND “trauma” between May 2021 and August 2021 using PubMed, Google Scholar, and inquiry of cited papers in existing narrative and systematic reviews as well as meta-analyses on similar topics. Additional articles were added through updated searches, new publications, and recommendations from reviews between September 2021 and September 2026. Titles were screened for duplicates, and abstracts were screened to ensure the inclusion of an art therapy program or experiential for any type of trauma exposure or traumatized person/population. Papers were included regardless of age, sex, race/ethnicity, trauma type, or related psychiatric outcomes of represented patients/participants. Papers that were unavailable in English, used other creative arts modalities like dance/movement therapy, yoga/mindfulness, narrative medicine, or music therapy, or used art as therapy but not art therapy were excluded. Given the current body of work, which has limitations in terms of sample sizes and a paucity of randomized controlled trials, systematic reviews, and meta-analyses, we call for a larger and more rigorous evidence base.

## 2. Neuroscientific Basis for the Use of Art Therapy

Theoretical understanding and preliminary evidence supports the hypothesis that art therapy has the capacity for engaging and therapeutically modulating brain networks (J. King et al., 2019), including reward pathways (Kaimal et al., 2017; Kaimal et al., 2022), executive function (Belkofer et al., 2014), and regulation (M. S. Walker et al., 2018). Art therapy interventions focus on executive function, self-efficacy, community relationships, and mastery of challenges (Casey et al., 2019; Feder et al., 2019). Art therapists leverage creativity to integrate cognitive, sensory, and emotional processes in order to synthesize the conscious and subconscious (J. L. King et al., 2019). Kinesthetic exploration of media (e.g., cutting or tearing paper to create new images from (Feen-Calligan et al., 2020)) may facilitate a feeling of release, while sensory media can promote calmness (Hinz, 2009, 2019). Art making is also hypothesized to engage brain regions responsible for accessing memories and processing emotions so that individuals can recall traumatic experiences and emotions in a safe environment while strengthening their sense of self (Hass-Cohen et al., 2018; Naff, 2014; Talwar, 2007). Individuals partaking in art therapy are able to distance themselves from emotion to create meaning through their artwork, ultimately allowing for a “helpful reconstruction of the experience” (Naff, 2014; Schouten et al., 2015). Often, art therapists, who are licensed and/or registered (depending on region of practice) clinicians (some with board certifications), will implement art therapy alongside other treatments, and art therapy can be an effective option when other interventions are not helping the individual (Schouten et al., 2015). Art therapy can also be combined with other expressive therapies (e.g., music therapy; dance/movement therapy), narrative, and mindfulness-based approaches (Kalmanowitz & Ho, 2017; Schweitzer et al., 2013; Ugurlu et al., 2016). For youth, art therapy can provide a safe modality for sharing their stories (Akthar & Lovell, 2019)—especially for those who may face communication barriers in the treatment space due to developmental conditions, language barriers, and selective mutism (perhaps in the wake of abuse and other traumas). Art therapy offers the potential to engage youth, help them feel a sense of control and structure, and reduce stress through the manipulation of art media and the creative process (Ugurlu et al., 2016). Here we review the supporting literature to date across the Hierarchy of Evidence, for adults and youth.

## 3. Research Across the Hierarchy of Evidence

With each level of the Hierarchy of Evidence, rigor increases. Theory and methodology define the foundational level, providing a basis for clinical application and research. Case and cohort studies contribute to the understanding of implementing various art therapy interventions, while randomized control trials (RCTs) and meta-analyses at the top of the hierarchy analyze the effects of art therapy on large populations. There are an abundant number of case studies in the art therapy field—these are critically important to the development and testing of experientials and make valuable contributions to the interventions themselves. Stemming from this work is a growing body of larger population empirical studies, randomized controlled trials, and, resultantly, meta-analyses to further increase usage of and support for art therapy.

### 3.1. Individual Case Studies

#### 3.1.1. Adults

Art therapy can be leveraged for both assessment and therapeutic purposes in the trauma treatment space. In terms of assessment, one case study used scribble drawings to identify guiding treatment themes and goals for a survivor of childhood sexual assault (CSA; Tripp, 2007). The client was then guided to illustrate traumatic memories while concurrently listening to quiet sounds via headphones that alternated between each ear and coincided with small vibrations on each knee (combining practices from eye movement desensitization and reprocessing [EMDR] during the art therapy session). The auditory and tactile stimulation the client received during the session helped to focus and relax the client as they expressed their memories and feelings through their artwork. As exemplified here, combining art therapy and EMDR may be an effective strategy for those for whom cognitive behavioral therapy has been ineffective (Sigal & Rob, 2021). To further illustrate the themes in their artwork, the client attached feelings to the various colors they used. These color–emotion associations aided the client in shifting negative beliefs about personal responsibility for their caregivers’ actions away from the self.

While Tripp (2007) did not use a formalized assessment but rather an art experiential to guide treatment themes and goals, art therapy assessments do exist. Common art therapy assessments include the Bridge Drawing (Darewych, 2014; Darewych & Campbell, 2016; Girish & Lev-Wiesel, 2024; Teneycke et al., 2009), Person-in-the-Rain (Carney, 1992; Lichtenberg, 2013; Verinis et al., 1974; Willis et al., 2010), and House-Tree-Person (Judd, 2014; Kato & Suzuki, 2016; Li et al., 2011; Rohail, 2015; Roysircar et al., 2017). However, some assessments (e.g., Bridge Drawing; Person-in-the-Rain) are limited by a lack of psychometric validity and reliability studies, even in cases where scoring systems have been specifically developed or applied (Au et al., 2024; Bania, 2009; Hinz, 2019); others (e.g., House-Tree-Person) may not be valid assessments of trauma-related psychopathology (Lin et al., 2022).

Having had previous positive experiences with art as a coping tool in childhood, one CSA survivor worked with an art therapist to create a series of images centered on their own abuse. Through these images, the client was able to process their grief through a concrete representation of their emotions and memories. This breakthrough allowed the survivor to work through their experiences and resultant grief, rather than avoiding painful memories and emotions. Through their art, the client found that their desire to destroy the pain they felt was self-destructive, ultimately allowing the client to learn to channel their anger in non-destructive ways and be more compassionate to themselves and others.

Other survivors of CSA have found strength and empowerment through clay. Working with clay can recreate implicit memories of touch, both pleasant and/or hurtful, for clients. Through handling the clay, clients are able to experiment with movement and discover what actions bring the greatest somatosensory relief. For one pregnant individual with a history of abuse, working with clay provided an outlet for frustration, instilled feelings of strength, and provided constancy and reliability as the client found sensory awareness and new cognitive insights (Elbrecht & Antcliff, 2014).

Finally, Stace (2014) described a series of six dolls created by a woman diagnosed with posttraumatic stress disorder resulting from family violence and CSA. Doll-making was selected to engage kinesthetic-sensory processes and ultimately foster meaning-making through the cognitive-symbolic use of dolls. Dolls were created over 18 weekly sessions. The client selected materials and techniques for doll-making that contributed to a visual narrative. The client ultimately described the dolls as representing key figures in her childhood, including herself. The doll-making allowed her to process experiences of sexual abuse, family violence, and their dynamics. This process resulted in the client feeling “better and happier” with increased self-acceptance, greater confidence, empowerment, and a more positive identity and sense of self.

Case studies of art therapy describe applications not only for survivors of CSA, but also for military trauma. Through 15 art therapy sessions over the course of two years, one active-duty service member was able to externalize hallucinations and trauma memories through mask-making, painting, and sculpture (M. S. Walker et al., 2017). Over the course of sessions, the service member reported less intense flashbacks, improved cognitive and emotional function, and increased relaxation. At the end of the sessions, the service member decided to continue practicing art therapy in their own life. This case study exemplifies how art therapy can foster resilience through mastery of a skill.

The selected case studies described indicate that art experientials can aid adult clients in concrete representation and externalizing of their traumatic feelings or memories, ultimately providing clients the strength to move forward. Additionally, art therapy in combination with EMDR promoted a positive shift in cognition, causing individuals to view themselves with greater compassion. Continued engagement in art therapy by one client and self-selection of art as a therapeutic modality by another support acceptability. In their book chapter on the ETC, Lusebrink and Hinz (2021) further describe case studies demonstrating the use of the ETC. Their examples illustrate that not all trauma survivors can be treated alike, yet regardless of differences in trauma histories or treatment approaches, they may be able to develop a more positive sense of self and coping mechanisms.

The remainder of the case studies in this section focus on youth aged 18 or younger. The youth and adult studies have been separated as it is important to recognize how youth may process trauma differently, and how their treatment plan may differ from that of adults.

#### 3.1.2. Youth

A case study of a teenage burn survivor illustrates a four-stage model design for trauma-informed art therapy: (1) dealing with the impact of one’s trauma and connecting the dots to where one is now, (2) building therapeutic alliance and channeling energy into creativity, (3) acknowledging what happened, emotional reactions, and overcoming barriers, and (4) reconstructing and fostering meaning (Appleton, 2001). In the first stage, the teen used black to represent their pain and yellow to represent their hope for the pain to be gone in their drawings that depicted the client’s feelings toward their transition to burn trauma. In the second stage, the teen became defensive of their situation as they focused their energy on creating. The individual drew with colorful pens during this stage, creating fantasy themes consistent with the denial of trauma in the unconscious. During the third stage, the teen acknowledged their feelings of anger and depression and created a self-portrait using more colors and energy than previous drawings. Within the self-portrait, the teen was able to express their anger and resentment but also drew a purple egg to represent rebirth upon anticipated discharge from the hospital. The final stage allowed the teen to discuss unresolved concerns as they prepared to leave the hospital. The teen drew a black box, as a safe container of their experience, with yellow rays radiating to represent their looming rebirth. Through these drawings, the teen was able to communicate their feelings while determining a sense of social identity. For this client, art therapy provided a safe outlet for the feelings of pain they experienced while seeking treatment in the hospital and allowed the client to engage in a comforting practice amid the various medical procedures endured.

Art therapy may be helpful for children who experience cognitive difficulties, including greater difficulty verbalizing their experiences. For example, one 11-year-old with Fetal Alcohol Effects (FAE) from Fetal Alcohol Spectrum Disorder (FASD) participated in a 9-week art therapy group with 18 individual sessions following the death of their mother by suicide and a history of physical and sexual abuse (Gerteisen, 2008). Drawings were used to externalize and communicate the child’s fears, feelings of helplessness, and feelings of immobility. The therapist then introduced the child to mandala drawings. The child was able to relate the mandala to a positive feeling and revisit previous drawings with representations of their fears to add the elements of the mandala. The client in this study was able to effectively express feelings of fear and inadequacy while finding a new perspective through their artwork. For children with contraindications to effective cognitive behavioral therapies, it may be necessary to provide a mechanism for them to process trauma nonverbally, as these client can more easily identify their feelings and explore possible resolutions through interventions such as art therapy.

Kruger and Swanepoel (2017) described how traumatic memories can be integrated with other life memories through digital collage making. In this example, four adolescent clients created digital metaphorical artworks during the middle stage of therapy as they were processing traumatic material. Collage making allowed them to attach multilayered meaning to memories, which led to a new understanding of how their traumatic experiences could be contextualized within their broader lives.

#### 3.1.3. Summary and Synthesis

In these case studies, art therapy allowed clients to shift from negative to more positive cognitions, consciously experience physical sensations while creating art in a safe space, separate self-identities from events in their past, and facilitate greater awareness and strength. Across studies in both adults and youth, clients frequently used colors to convey emotional meaning. While formal art therapy assessments exist as described, the ways in which clients used colors (as well as objects and layering of images through collage) to prescribe meaning and process emotions highlight the importance of debriefing with clients on the art that they create. Debriefing ensures that the process does not end with a created work but rather fosters a conversation for the therapist to better understand the client’s thoughts, emotions, and needs. Across the studies, being able to concretely represent emotions and memories was a mechanism through which individuals were able to process traumatic experiences. Clients could safely engage with painful emotions and memories; simultaneously, they found ways to experience and relate with positive emotions. The diversity of clients reflected in these case studies highlights that art therapy can benefit individuals across the lifespan and with various trauma histories; the diversity of art materials used highlights the importance of flexibility and adaptation for art therapists when working with clients to not take a one-size-fits-all approach.

While these studies are toward the bottom of the evidence hierarchy, case studies provide in-depth information on how each step of the art therapy process may affect both adult and youth clients. Larger-scale studies and clinicians may apply these methods and experientials in future research and their own practice.

### 3.2. Cohort Studies

Cohort studies allow for broader application, observation, and generalization of findings by working with groups rather than individuals. The group setting may confer the additional benefit of social support throughout the process, and individual resilience may be strengthened with the additional support of a social system. Positive social support can reduce one’s response to stressors, and social support can act as a buffer to posttraumatic stress disorder (PTSD; Sippel et al., 2015), a trauma-related disorder that occurs in approximately 7–10% of the general population exposed to trauma.

#### 3.2.1. Adults

Hass-Cohen et al. (2018) delineate a four-drawing protocol (separate and distinct from the four-stage model described above) that requires participants to draw (1) their problem, (2) themselves, (3) their internal and external resources to help with the problem, and (4) current self-perceptions. Participants may be asked to write short stories and create a title for each drawing. This model was applied with a predominantly female cohort who reported a decrease in negative affect, trends in pain reduction, and increased resiliency following a single-session implementation of the protocol (Hass-Cohen et al., 2018). Participants also gave positive ratings of the drawing activity. There was no significant change in posttraumatic growth and cognitions. Single-session studies have limitations, as they may cause state or momentary change, but not a trait or lasting change; however, single sessions are more scalable as they require less time and resources (Schleider et al., 2020b). Single sessions may also be sufficient to introduce individuals to coping skills that they can repeatedly implement at their own volition. Extensive research on single-session interventions based on cognitive behavioral therapy and parent management training principles for youth, caregivers, and healthcare workers has shown this method to be effective at scale (Jans et al., 2024; Schleider, 2024; Schleider et al., 2020a; Schleider & Weisz, 2017a, 2017b; Sotomayor et al., 2025). The four-drawing method may also be a beneficial art therapy intervention to use in multi-session art therapy protocols or alongside other methods of psychotherapy in the trauma treatment space.

Group interventions can also enhance accessibility for historically marginalized and neglected groups, like incarcerated individuals. (Hongo et al., 2015). Many women who are incarcerated have experienced sexual or physical abuse, assault, and domestic violence. The monotonous daily activities of persons who are incarcerated may create an institutional dependency and limit the individual’s sense of identity; art therapy provides an opportunity for expression, communication, cognitive improvement, and emotional processing. After completing their art experientials, five themes were observed across the artwork of 20 incarcerated women who participated in a 6-week group: the potential to dream, feeling connected, mutual understanding, release of feelings, and unselfish concern (Hongo et al., 2015). The sessions helped to develop group support, and on an individual level, participants were able to build a sense of peace and freedom within the workshops. Many participants expressed a wish for continued art programming. Having a creative outlet through this program was a positive experience for many of the women, and including forms of art therapy in prisons could improve outcomes post-incarceration.

Another group art therapy study applied a three-phase model across 11 h-long sessions for nine refugees and asylum seekers with a history of childhood trauma and diagnosed PTSD (Schouten et al., 2019). Phase I was stabilization and symptom reduction, consisting of art experientials aimed at reducing stress and increasing the individual’s sense of control (e.g., collages and drawings to create a safe space or positive images). Phase II was trauma-focused—in each session, participants were asked to translate a positive or traumatic memory into artwork. Phase III was integration and meaning-making. Participants focused their artwork on combining the future with past memories and emotions they had previously expressed. After completing the sessions, participants reported decreased feelings of stress, worry, avoidance, and intrusive thoughts, and increased feelings of relaxation and the ability to look at the future confidently. The participants were able to express memories and emotions through their artwork that they were unable to express before, and one participant shared that “art therapy felt better than talking alone.”

A group study on war veterans analyzed common themes that emerged in veterans’ art after returning from combat (Tomassoni & Galetta, 2016). During the group sessions, participants were asked to draw free associations with any colors, then discuss their drawings in the group. Veterans tended to depict battle scenes with killed or wounded combatants, and oftentimes the artist chose red and black colors in their drawings. War-related themes often occurred, as well as illustrations of nightmares and graves. Many participants reported feelings of horror, sadness, devastation, and pain. Group support during the sessions aided many participants in feeling as though they were accepted again, effectively reframing the veterans’ self-perceptions. Group sessions also allowed veterans to observe their experiences from a new perspective and give new meaning to such experiences.

#### 3.2.2. Youth

Multiple youth cohort studies indicate the potential promise of art therapy for reducing posttraumatic stress, anxiety, conduct problems, and emotional problems in youth resettled as refugees exposed to civilian war trauma and forced migration (Burruss et al., 2021; Feen-Calligan et al., 2023a, 2023b, 2020). Introductory activities that foster a sense of safety within the group are recommended; for example, in one study, participants created a large puzzle in which participants were able to draw something about themselves or a feeling on their puzzle piece. Additional recommended art interventions include creating trees out of paper bags in which participants represented their personal strengths on the trunk of the tree, and resources they could reach for on the branches; watercolor paintings; collage; depicting personal stories through puppetry. Focused breathing and mindfulness practices in tandem can help youth reduce stress and center themselves to fully engage in the art therapy experience. Offering alternative art activities is important—choice can be critical in the art therapy process, especially if potentially aversive media are to be used (e.g., some individuals are not amenable to clay due to sensory preferences). Not only children, but also their parents, report positive impacts as a result of such programs.

Similarly, art therapy can support groups of youth exposed to mass trauma, like the 9/11 terrorist attacks (Testa & McCarthy, 2004). In this study, youth created a mural of New York City (the location of the attack). While creating the art piece, the youth discussed their feelings, particularly their feelings of vulnerability, concerns about safety and the loss of life. Through the mural, youth were able to create a narrative of their trauma and find a way to tolerate the presence of the traumatic memory. The themes in the mural were solidarity and unity, and after finishing the mural, the group members felt a sense of pride. Group mural making is a therapeutic modality in and of itself (Rossetto, 2012; Wilkie, 2018) that may have meaningful applications for community-based therapy and especially in fostering collectivism (Chiao et al., 2009; Triandis, 1988).

Just as art therapy may be integrated with psychotherapeutic practices (e.g., EMDR), art therapy may also be combined with other expressive modalities. For example, Ugurlu et al. observed improvements in PTSD, anxiety and depression symptoms over the course of a five-day art, music, and dance therapy workshop for Syrian refugee children who resettled in Turkey (Ugurlu et al., 2016). The art interventions facilitated youths’ discussion of their traumatic experiences, sharing emotions and thoughts, and identification of their emotions to gain insight into their hardships. Quinlan, Schweitzer, Khawaja and Griffin (Quinlan et al., 2016) conducted a similar trial of a 10-week art therapy protocol that included a range of art interventions (Quinlan et al., 2016). The authors found reductions in behavioral and psychological problems, but the means of assessing the children’s symptoms had not been validated for youth, cautioning interpretation of the findings. In Montreal, Quebec, Canada, creative arts programming for refugee children facilitated expression of emotions and increased self-esteem through visual arts, singing, puppets and other group activities (Rousseau et al., 2003, 2005; Rousseau & Heusch, 2000). The children were able to create “a meaningful personal world while simultaneously strengthening the link of the child to the group” by utilizing myths originating in their homelands in their creative process (Rousseau et al., 2005). Lastly, 30 refugees aged 11–20 from Burma or Thailand, who had been resettled for five years, participated in a school-based art therapy program for a single semester (Rowe et al., 2016). Over the program period, participants reported improvements in anxiety symptoms and self-concept.

#### 3.2.3. Summary and Synthesis

In these cohort studies, individuals expressed their emotions and experiences through their art. Notably, many cohort studies were designed for specific groups of trauma-exposed individuals: women who were incarcerated, persons forcibly displaced due to war, war veterans, and survivors of terrorism. The shared experiences within the cohorts allowed participants to feel additional support and more empowered to discuss emotions and process traumatic events with one another—both verbally and through art. Group art therapy interventions could scalably provide support in the aftermath of mass trauma and foster social connectedness critical to recovery. Group-based experientials (e.g., mural-making, debriefing as a group) can further foster social connectedness. However, even where statistically significant improvements in self-reported measures of trauma-related symptoms and other phenotypes were reported, most of these studies lacked control groups. As a result, it is unclear whether the effects were due to the intervention specifically or to the effect of time. Additionally, willingness to join and participate in open-arm studies or single-arm studies may introduce sampling bias and biased findings, such that individuals who are motivated to participate in treatment studies may be more likely to respond. These limitations can be addressed through randomized controlled trial designs.

### 3.3. Randomized Controlled Trials

Randomized controlled trials (RCTs) serve as the gold standard method for evaluating the efficacy of various treatments. While the nature of behavioral interventions does not permit double-blinded trials as pharmaceutical interventions do, the ability to compare modalities with one another as well as change over time not due to active treatment is meaningful.

#### 3.3.1. Adults

One RCT for motor vehicle accident (MVA) survivors (Wang et al., 2015) described positive change over time observed in PTSD symptom severity in both the art therapy and waitlist control groups, and no significant differences between the groups in regard to the severity of anxiety and depression twelve months post-treatment. Another trial for students with a variety of trauma exposures compared art therapy to an active control—narrative therapy (Pizarro, 2004). Narrative therapy provides individuals with numerous benefits such as reducing stress, promoting coping skills, and improving overall health. However, writing—particularly about traumatic events—may also provoke some level of anxiety (although this may be beneficial for activating representational structures and driving positive change through exposure) (Grasser, 2025). For some individuals, linguistic/literacy barriers may preclude implementation and efficacy of narrative methods. Participants in the Pizarro (2004) study were randomly assigned to either write about stressful or traumatic events, draw about stressful or traumatic events, or draw a still life (control) over two hour-long sessions. A significant decrease in social dysfunction was observed for the narrative therapy group relative to the art therapy group. Art and narrative therapy groups did not differ in terms of positive changes observed on measures of anxiety, insomnia, and depression, indicating that both modalities may be effective for alleviating some trauma-related symptoms (Pizarro, 2004). Finally, a third art therapy group for veterans found that art therapy not only relieved negative symptoms but also facilitated positive gains—veterans who participated in an art therapy group had an increase in humor after treatment compared to control (Gilgoff et al., 2020).

Abbing et al. (2018) summarized three RCTs involving students with PTSD symptoms, students with exam anxiety, and prisoners with pre-release anxiety. The diversity of samples reflects the broad applications of art therapy, yet the paucity of RCTs from a search of thirteen databases and two journals reflects a need for more research on art therapy’s efficacy across diverse populations and needs. The studies identified leveraged trauma-related mandala design, collage making, free painting, clay work, still life drawing, and House-Tree-Person drawing. Summarized evidence suggested that art therapy induced relaxation, facilitated access to unconscious traumatic memories, and improved emotion regulation.

#### 3.3.2. Youth

One RCT in youth to date has described a specific program, the Chapman Art Therapy Treatment Invention (CATTI), for treatment of PTSD in pediatric patients (Chapman et al., 2001). The study included 7–17-year-olds admitted for traumatic injuries that required a minimum of 24 h of hospitalization. The CATTI allows the engagement of visual and verbal modalities, ultimately facilitating the transference of nonverbal memories to a linguistic narrative. No significant difference in the reduction in PTSD symptoms was observed between CATTI treatment and control groups; however, the participants who received the art therapy treatment showed a reduction in acute stress symptoms.

#### 3.3.3. Summary and Synthesis

As stated above, an inherent limitation of behavioral interventions is the lack of blinding; for clinical trials (and other designs) writ large, bias due to the treatment-seeking nature of the study sample is also important to consider. Do individuals self-select to participate in art therapy studies, or trials in which they may receive art therapy, because they enjoy creative arts experiences? How might past experiences in arts engagements bias outcomes, and how strong of a role does patient preference play in treatment outcomes? While some of these biases reflect inherent design limitations, researchers should consider inclusion of assessments that measure credibility and expectancies surrounding interventions—that is, how logical and successful an individual believes a particular intervention to be and how much they feel that it will help them to improve their functioning. Further, the lack of RCTs in trauma-exposed youth reflects a critical gap in the field, particularly given that up to 80% of affected youth do not receive treatment, and of those who do, up to 50% fail to respond to gold-standard psychotherapies and medications.

Clearly, there is significant work to be done in the application of randomized controlled trials to evaluate not only art therapy but also other expressive art therapy methodologies. Ultimately, the goal of such trials should not be to show superiority of art therapy to other trauma treatment methods, as the intention is not to replace existing approaches. Rather, the goal is to support the inclusion of art therapy in the treatment toolkit and provide necessary evidence to ensure safety, accountability, and facilitate insurance coverage as well as provider referrals.

### 3.4. Systematic Reviews and Meta-Analyses

At the uppermost level of the Hierarchy of Evidence, systematic reviews and meta-analyses facilitate the most meaningful comparisons and potential to make stronger conclusions. Additionally, they can identify gaps in the literature and key outstanding questions for future research.

#### 3.4.1. Adults

Schouten et al. (2015) summarized 6 studies and found that across half of the studies, psychological symptoms significantly decreased in art therapy groups compared to control. The studies were limited by small samples, predominantly undergraduate and non-population-representative samples, and the variation in art experientials, treatment duration, and use of combination therapies. The authors concluded that art therapy in combination with other psychotherapeutic modalities resulted in the most meaningful reductions in symptom severity.

Bowen-Salter et al. (2021) conducted a systematic review of 44 studies to determine standard elements of trauma-focused art therapy. On average, the duration of sessions was an hour to 90 min, and more than half of the studies reported that participants continued to practice art therapy to some extent after the sessions concluded. Twelve of the 44 studies designed their study using existing models of art therapy, and only 16 studies had standardized outcome measures for their data and results. The author concluded that studies need to more clearly define the qualifications of the art therapist leading the sessions and that art therapy protocols employed in studies cannot be generalized beyond that case. Additionally, there is no standardized protocol for using art therapy as treatment, which can be a future focus in the art therapy community.

A meta-analysis of 19 studies sought to determine under which conditions (age, symptoms, treatment length, group or individual therapy) art therapy has the most significant effects (Campbell, 2009). The author concluded that (1) art therapy is an effective treatment for reducing anxiety-related symptoms, (2) art therapy is equally effective in children and adults, and (3) outcomes do not differ whether therapy occurs in the group or individual setting.

#### 3.4.2. Youth

Eaton et al. (2007) examined the use of art therapy for traumatized children across 12 studies. Art therapy was leveraged for children who have experienced physical and sexual abuse, children exposed to war and gun violence, those who experienced the 9/11 attacks, and those who have lost a loved one. Duration of treatment ranged from weeks to years. Authors concluded that art therapy facilitated the relationship between the child and therapist through the combination of art making and storytelling. The authors found that current research regarding art therapy and childhood trauma cannot be easily generalized for a multitude of reasons: (1) many studies did not provide enough information about the art therapy methodology or the formal structure used; (2) most studies are case studies rather than cohort studies and RCTs; and (3) there is a lack of case–control studies for art therapy due to the ethical concerns that one group of children would not receive treatment. As described elsewhere, stepped-wedge cluster randomized controlled trials offer a solution to ethical concerns by providing a design that allows testing of time versus intervention effects while also ensuring that all study participants receive treatment in a timely manner (Grasser & Marusak, 2023).

In a broader systematic review of psychosocial interventions for refugee youth exposed to trauma, mixed effects of expressive art therapies were identified across 13 studies (Cowling & Anderson, 2023). The authors highlighted interventions that utilized multiple modalities as producing positive improvements, and improvements in symptoms were observed from preschoolers through adolescents without significant effects of geographic location, country of origin, and mode of delivery. Finally, a recent meta-analysis of RCTs and quasi-experimental studies of creative arts-based interventions in youth ages 3–18 (Applewhite et al., 2025) indicated strong, significant effects of creative arts-based interventions on PTSD and other trauma-related symptoms, particularly in West African and Middle Eastern samples, compared to Western samples.

#### 3.4.3. Summary and Synthesis

Summarizing not only findings from systematic reviews and meta-analyses, but also our own review herein, the current evidence indicates that (1) art therapy can be applied for a variety of trauma-exposed populations across the lifespan, (2) group designs may be particular impactful in the context of shared traumatic experiences and mass traumatic events, (3) consistency in provider qualifications and training is needed to more robustly evaluate the effects of art therapy interventions and integrate mounting evidence, and (4) while standardized protocols would support synthesis of outcomes, such protocols should be developed with flexibility in mind to offer alternatives for individuals who may respond negatively to specific media or may prefer media that differentially engage kinesthetic-sensory needs of the individual (be they subconsciously or consciously aware of these needs).

There is a great deal of work to be done within the art therapy field to manualize trauma-informed art therapy practices and conduct clinical trials in both ethical and scientifically rigorous manners. Critically, the substantive increase in art therapy research in the 2000s as compared to the 1900s, coupled with trauma being one of the most central themes, according to a bibliometric analysis of art therapy, indicates the substantive motivation of clinicians, researchers, and affected individuals towards this goal (Liu et al., 2022). This can be supported by increased funding for such research, peer review in rigorous, high-impact journals, and broader socioeconomic support for creative arts and movement-based interventions. Further, legislative action to support licensure of art therapists can ensure necessary qualifications are met and that findings of individual studies are not specific to one provider or protocol.

## 4. Discussion

Despite the literature providing preliminary support for the effectiveness of art therapy in trauma recovery, the evidence base remains limited and methodologically heterogeneous (Abbing et al., 2018; Strazdiņa et al., 2026, p. 2). Findings from across the Hierarchy of Evidence indicate that art therapy can be applied with various populations experiencing trauma (Howie, 2015), including adults and youth, war veterans (Howie, 2017), refugees, and those who have experienced CSA. For many, art therapy provides a safe environment in which one may explore their past trauma and emotions and allow for externalization of the trauma (Grasser & Jovanovic, 2021). Participants may engage in individual sessions, working directly with a credentialed professional [i.e., ATR-BC—registered, board-certified art therapist (Art Therapy Credentials Board, USA)] who may also be jointly licensed/certified in counseling psychology, clinical psychology, social work, or other clinical profession. An art therapist may lead group sessions for clients to create art while feeling supported by others with similar experiences—potentially maximizing therapeutic gains in accordance with social safety theory (Slavich, 2022). While the published evidence base may be less comprehensive for art therapy compared to other treatment approaches like CBT, cognitive processing therapy, pharmacotherapy, and others, in practice art therapy is used for treatment of trauma-related symptoms in private practice clinics, Veterans Affairs hospitals, residential treatment settings, hospital settings, and beyond. Thus, patients and clinicians may work together to consider art therapy as an adjunctive intervention, an integrative approach, or primary treatment method for trauma-related symptoms. However, widespread clinical use should not itself be interpreted as evidence equivalent to that supporting more extensively evaluated interventions—meaning that the call for more rigorous and robust research on art therapy in the trauma treatment space would be meaningful for ensuring safety, prompting insurance coverage and referrals, and expanding the application of art therapy.

“The Expressive Therapies Continuum (ETC) remains one of the most influential frameworks for understanding how sensory, affective, cognitive, and symbolic processes are expressed and integrated in art-making” (Strazdiņa et al., 2026, p. 2). However, the ETC has largely remained a descriptive framework. We can see tenets from the ETC reflected across case and cohort studies that used iterative approaches: first, the individual or group is gradually introduced to the process of art therapy and art making while building trust in the environment. Then, the therapist and individual may work together to identify a traumatic memory, problem, or difficult emotion that gives rise to naming resulting feelings and responses. The individual can in turn examine themselves within the context of the event, their response to it, and how it fits into their life now to find a way to process and move forward. These steps align with the ETC: an individual may become aware of an internal state that signals a difficult memory, problem, or emotion (kinesthetic-sensory), label how that makes them feel or how they respond to it (perceptive-affective), and make meaning out of it through cognitive understanding and insight (cognitive-symbolic). Children who partake in art therapy will typically learn coping strategies in preparation for revisiting traumatic memories and are then able to find posttraumatic growth, while adults may identify their traumatic memories first and then find ways to cope through art making. The art products provide a tool through which the client and the therapist can have a discourse. The therapist may ask the client about the colors, pressure, perseveration, symbols, and other aspects of their artwork to better understand the individual, their experiences, and their emotional state.

The kinesthetic, sensory nature of various art experientials can provide the opportunity to engage the whole body through this process, with therapeutic goals that include discharging energy, providing relaxation, encouraging rhythm and self-soothing, and reconnecting with the wisdom of the body (Hinz, 2019, p. 49). Collaging, painting, and drawing are common experientials identified through this review, facilitating the creation of images that express emotions and traumatic memories. For example, collage can be used to engage participants in storytelling and personal expression while also integrating kinesthetic and sensory experiences. Cutting or tearing paper may be stress-relieving and selecting images and arranging pieces can encourage calmness and promote storytelling (Feen-Calligan et al., 2020). Visual representations of trauma are a helpful tool for individuals to communicate their thoughts, particularly for individuals who are either unable or uncomfortable communicating themselves verbally or through writing (Pizarro, 2004).

Colors may provide an art therapist with insight into an individual’s feelings, or other information about the artist. Clients incorporate colors differently depending on their experiences—for the client who experienced CSA, the color red was used to represent the client’s anger and black was used to express depression (Tripp, 2007). When a veteran used red and black, the colors depicted violence in a battle scene (Tomassoni & Galetta, 2016). The color red was used in a more negative context in both scenarios; art therapists can consider how the choice and utilization of color in an individual’s artwork may be influenced by an individual’s traumatic experiences. Artwork that includes bright colors might refer to a sense of hope (e.g., M. S. Walker et al., 2016), alluding that brightness is coming, or adding energy to the artwork. Of course, color is idiosyncratic, and there are different cultural uses of color. With this in mind, art therapists may mindfully observe patterns in clients’ use of color. Exploring use of color with clients can contribute to greater awareness and insight into their representations of emotions and can also provide evidence of an individual’s change and progress.

Images may be unique to an individual’s trauma as well. As mentioned in this review, veterans often depict battle scenes and faces to reflect the setting of their traumatic memories (Tomassoni & Galetta, 2016; M. S. Walker et al., 2016). Making paper mâché masks allows veterans to create an externalized version of themselves or memories of individuals they remember from their time in combat (M. S. Walker, 2017; M. S. Walker et al., 2017; R. L. Walker et al., 2014). Both clients with CSA and children may include images of monsters in their artwork, yet for clients with CSA the monsters might represent their assaulters, while the monsters that other children draw might represent their fears (Gerteisen, 2008; Sigal & Rob, 2021). In this review, the artwork of incarcerated women relates to their potential to dream, perhaps alluding to the possibilities after incarceration, and feeling connected with other members in the group (Hongo et al., 2015). Artwork created by refugees is often impacted by themes of the future as a way to focus on new beginnings.

In the wake of the COVID-19 pandemic, emerging evidence also supports the application of art therapy in the virtual treatment space (Grasser & Javanbakht, 2021). Relatedly, healthcare providers and first responders who have been affected by secondary traumatic stress (both during the COVID pandemic and other global disasters) may benefit from art therapy, as previous research has supported art therapy for management of secondary traumatic stress and burnout (J. King et al., 2019).

While a wide range of the literature describing the theoretical foundations, case studies, and cohort studies of art therapy is available, more research is still needed to develop standardized protocols and to manualize trauma-informed art therapy. Referring back to the hierarchy of evidence, the next phases of research for art therapy should include more randomized controlled studies, subsequently resulting in systematic reviews and meta-analyses. The majority of studies summarized herein were limited by small samples (mostly ranging from one to 30 participants per study), heterogeneous interventions, inconsistent outcome measures, absence of long-term follow-ups, and therapist variability. RCT designs can address these concerns, with inclusion of active control groups or stepped-wedge designs to make sure all participants receive some form of treatment. The stepped-wedge design offers a valuable opportunity to both control for the effect of time and address concerns regarding all participants receiving intervention. The latter has previously precluded waitlist control or no-treatment control designs in the art therapy intervention space. RCTs should compare art therapy to pharmacotherapy, CBT, CPT, or other psychotherapeutic methods, or combination treatment, for specific diagnoses (e.g., PTSD, acute stress disorder) or for transdiagnostic treatment with appropriate power considerations. Pre-registering hypotheses or publishing protocol papers is a proactive step to avoid publication bias. Additionally, in a few studies, such as the study testing the effects of art therapy on individuals who have been incarcerated, there is a wish to continue the art therapy programs and practices. This creates another focus for the future of art therapy—providing communities with the resources to either continue art programs for individuals or implement community-based programs. Due to the high acceptance of the ETC by the art therapy profession, incorporating the ETC in the theoretical framing, methodological design, and analysis of art-based interventions and research is a recommended step. Theoretical mechanisms and preliminary evidence suggest that art therapy may reduce trauma-related symptomatology, promote resilience, and strengthen connections across sensory, attention, and regulatory networks. However, replication studies are necessary to confirm initial theories and hypotheses.

While a singular narrative review cannot fully reflect the diversity and richness of art therapy or types of traumatic stress in different societies and situations, we hope that this article can help guide future research. Considering that cohort studies indicated a positive role of the group structure for mass trauma and considering that our overall narrative review highlighted benefits for individuals across the lifespan, family-centered art therapy programs should be manualized and tested in future RCTs. Broadly, manualization and publication of art therapy protocols would allow for synthesis and direct comparison of studies across the hierarchy of evidence and across populations. Yet manualization should not hinder adaptation, as we have highlighted the diverse needs of clients who engage with art therapy related to materials and experientials.

## 5. Conclusions

Decades’ worth of experience and expertise, as well as thousands of years of human tradition, support the hypothesis that art therapy equips individuals with tools for expressing emotions and better processing events (both daily stressors and past traumatic memories). The peer-reviewed evidence base supporting the application of art therapy for the treatment of trauma-related psychopathology primarily comes from small case studies and cohort studies that are underpowered, lack control groups, and require replication. The quality of evidence supporting art therapy for trauma treatment can be enhanced by more standardization, manualization, and rigorous randomized controlled trials with larger samples. Such research is critical, as clinicians could consider incorporating art as therapy in their practice to facilitate the identification, processing, and reframing of traumatic memories through drawing/painting, mask making, and collage. Providers could also work in tandem with or refer clients to art therapists to complement other forms of psychiatric care. Finally, art therapy interventions are readily scalable and could be implemented at the school and community level to build resilience across ecological systems and mitigate the development or worsening of psychopathology.

## Data Availability

No new data were created or analyzed in this study. Data sharing is not applicable to this article.

## Abbreviations

The following abbreviations are used in this manuscript:

ETC	Expressive Therapies Continuum
PTSD	Posttraumatic Stress Disorder
CSA	Childhood Sexual Assault
EMDR	Eye Movement Desensitization and Reprocessing
FAE	Fetal Alcohol Effects
FASD	Fetal Alcohol Spectrum Disorder
RCT	Randomized Controlled Trial
MVA	Motor Vehicle Accident
CATTI	Chapman Art Therapy Treatment Intervention
ATR-BC	Registered, Board Certified Art Therapist
CBT	Cognitive Behavioral Therapy
